# Vaccination, regular exercise, and prevention of chronic lung disease reduce exacerbation of COVID-19 severity in northern Okinawa, Japan: A cross-sectional study

**DOI:** 10.1265/ehpm.23-00281

**Published:** 2023-11-25

**Authors:** Takuji Kishimoto, Daisuke Tasato, Yoshitaka Nagasawa, Yuri Higure, Michika Setoguti, Rin Tibana, Akihiro Yamashiro, Tatsuya Miyazato, Hayashi Shokita

**Affiliations:** 1 Department of Health Screening, Okinawa North Medical Association Hospital, 1712-3 Nago City, Okinawa 905-8611, Japan; 2 Department of Respiratory and Infectious Diseases, Okinawa North Medical Association Hospital, 1712-3 Nago City, Okinawa 905-8611, Japan; 3 Department of Endocrinology, Metabolism and Dialysis, Okinawa North Medical Association Hospital, 1712-3 Nago City, Okinawa 905-8611, Japan; 4 Department of Gastroenterology, Okinawa North Medical Association Hospital, 1712-3 Nago City, Okinawa 905-8611, Japan

**Keywords:** COVID-19, Severity, Lifestyle, Vaccine, Regular exercise, Chronic lung disease, Cross-sectional study, Target trial emulation

## Abstract

**Background:**

As at June 14, 2023, the coronavirus disease 2019 (COVID-19) pandemic had affected more than 767 million people and caused more than 6.9 million deaths worldwide. This study aimed to clarify the lifestyle factors that influence the exacerbation of COVID-19 severity.

**Methods:**

This was a cross-sectional study of patients with COVID-19 whose severity classification of “moderate or severe” (COVID-19 exacerbation) was defined as an objective variable. The 1,353 participants were selected from 4,899 patients with COVID-19 between August 10, 2020 and December 10, 2022. Participants who underwent a specific health checkup before the date for a COVID-19 consultation were included. Using binominal logistic regression analysis, we evaluated the odds ratios (ORs) for COVID-19 exacerbation according to lifestyle-related factors. Limitations were discussed using a target trial emulation framework which clarifies problems in observational studies.

**Results:**

The explanatory variables extracted as factors that exacerbated COVID-19 severity were gender (OR [man vs. woman]: 2.533, 95% confidence interval [CI] 1.484–4.322); age (OR [50s vs. 10s, 20s, or 30s]: 4.858, 95% CI 2.319–10.177; OR [60s]: 9.738, 95% CI 4.355–21.777; OR [70s + 80s + 90s]: 8.327, 95% CI 3.224–21.507); and comorbid chronic lung disease (OR [‘yes’ vs. ‘no’]: 2.892, 95% CI 1.227–6.818). The explanatory variables extracted as factors that reduce the severity of COVID-19 were hospital consultation year (OR [2022, predominantly Omicron variant prevalent vs. 2020, predominantly Alpha variant prevalent]: 0.180, 95% CI 0.058–0.559); number of vaccinations (OR [2 doses vs. 0 or one doses]: 0.223, 95% CI 0.114–0.436; OR [≥3 doses vs. 0 or one doses]: 0.090, 95% CI 0.035–0.229); regular exercise (exercising ≥2 days/week ≥30 minutes each at an intensity that causes a slight sweat for ≥1 year) (OR [‘yes’ vs. ‘no’]: 0.458, 95% CI 0.242–0.866).

**Conclusions:**

These results suggest the importance of vaccination, regular exercise, and prevention of chronic lung disease as measures against exacerbation of COVID-19 severity.

## Introduction

According to World Health Organization data available on June 14, 2023, the COVID-19 pandemic has affected more than 767 million people and caused more than 6.9 million deaths worldwide [1]. Attenuation of mutant strains, vaccination, and the development of treatments are reducing the health problems caused by COVID-19. However, clarifying the lifestyle factors that influence the exacerbation of COVID-19 severity is extremely important for managing the care of older people and people with comorbidities, as well as managing potential new virulent variants. The development of effective vaccines and therapeutics is also extremely important. Clarifying lifestyle risk factors and establishing countermeasures that emphasize lifestyle-related factors may help to mitigate symptoms even if infection occurs.

Many factors have been reported to exacerbate the severity of COVID-19, including older age, man gender, underlying comorbidities such as hypertension; diabetes; chronic lung diseases; heart, liver and kidney diseases; tumors; clinically apparent immunodeficiencies; and pregnancy [2–11]. Unhealthy diet and smoking are lifestyle risk factors [12–15]; however, lifestyle habits have not been sufficiently examined. Therefore, we conducted a cross-sectional study of patients with COVID-19 with the aim of clarifying the lifestyle factors that influence the exacerbation of COVID-19 severity.

## Methods

### Study design and population

This study was conducted at Okinawa North Medical Association Hospital that is one of the core hospitals in northern Okinawa Prefecture, Japan. This was a cross-sectional study of patients diagnosed with COVID-19. From August 10, 2020 to December 10, 2022, patients who had undergone a specific health checkup at our hospital before the date that they presented to the hospital for a consultation were included in this study.

### COVID-19 severity classification

Severity classification was performed based on the “New Coronavirus Infectious Disease COVID-19 Clinical Practice Guide” supported by the Fiscal 2020 Health, Labor and Welfare Administration Promotion Survey Subsidy. Briefly, the severity classification describes “asymptomatic” as a positive test for SARS-CoV-2 using virological testing but no symptoms consistent with COVID-19; “mild” as an oxygen saturation of ≥96% or clinical status of no respiratory symptoms or cough only, with no dyspnea; “moderate I” as an oxygen saturation of between 93% and 96% or clinical status of no dyspnea, no evidence of pneumonia, or respiratory failure; “moderate II” as oxygen saturation of ≤93% or clinical status of respiratory failure requiring supplemental oxygen; and “severe” as a clinical status of intensive care unit (ICU) admission or ventilator required. In this study, severity was classified into two categories: “asymptomatic or mild” and “moderate or severe.”

### Specific health checkup

Since April 1, 2008, a specific health checkup has been conducted in Japan under the initiative of the Japanese Ministry of Health, Labour and Welfare. The checkup focuses on preventing metabolic syndrome and cardiovascular disease. Specific health checkups are conducted once a year for people aged 40 to 74 years who have medical insurance.

### Objective variable

The severity classification in patients with COVID-19 was divided into two groups (“asymptomatic or mild” and “moderate or severe”) and analyzed. The objective variable was the severity classification “moderate or severe” (COVID-19 exacerbation).

### Explanatory variables

Explanatory variables obtained at the time of consultation were gender, age, hospital consultation year (2020, 2021, and 2022: predominantly Alpha, Delta, and Omicron variant prevalent, respectively), number of vaccinations, body mass index, smoking habit, comorbidities (including hypertension, dyslipidemia, diabetes, chronic lung disease, chronic kidney disease, heart disease, and malignant tumor), and immunosuppression status. Comorbidities and immunosuppression status were captured by history of current disease during treatment. Chronic lung disease includes chronic obstructive pulmonary disease, asthma, interstitial lung disease, etc. Chronic kidney disease includes renal disorders that persist for 3 months or longer and are under treatment. Immunosuppression status include organ transplantation or autoimmune diseases, and those receiving immunosuppressive therapy.

The vaccines administered to the participants were the mRNA vaccines BNT16262 (Pfizer/BioNTech) or mRNA-1273 (Moderna). Vaccinations during this research period were carried out five times based on national guidelines. The 1st to 3rd rounds were held in 2021, and the 4th and 5th rounds were held in 2022.

Explanatory variables obtained from the results of the most recent specific health checkup before the hospital consultation for COVID-19 included the following lifestyle-related items: (1) “having an evening meal 2 hours or less before bedtime 3 days or more per week,” (2) “eating snacks and sweet beverages outside of breakfast, lunch, and dinner,” (3) “eating faster than others,” (4) “skipping breakfast at least three times a week,” (5) “walking for at least 1 hour per day or having equivalent physical activities,” (6) “exercising at least 2 days per week for at least 30 minutes each session at an intensity that causes a slight sweat for at least 1 year,” (7) “frequency of drinking,” (8) “regular smoker,” and (9) “feeling refreshed after a night’s sleep”.

### Adjusted variables

The adjusted variables were all explanatory variables, which were applied to the binominal logistic regression analysis and analyzed using backward stepwise regression.

### Statistical analysis

Applying explanatory variables to the binominal logistic regression analysis, the odds ratios (ORs) of the explanatory variables were calculated using the forced entry method and backward stepwise regression. An α value of 0.05 was used to determine the significance of the analyses and variables. All statistical analyses were performed using IBM SPSS Statistics version 28 (IBM Corp., Armonk, NY, USA).

## Results

**Fig. 1:**
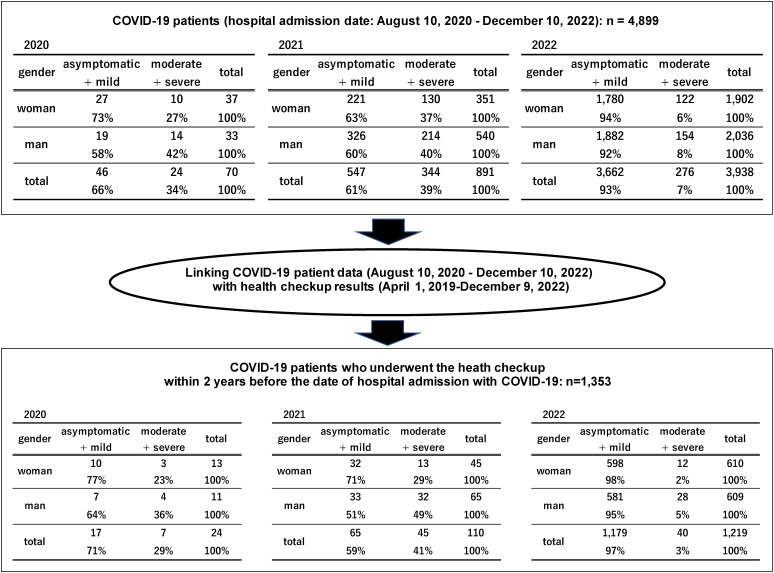
Selection of study population and severity classification by year and sex

During the study period, 4,899 patients with COVID-19 visited our hospital for a consultation. Of these, the 1,353 patients who underwent a specific health checkup at our hospital before the date that they visited it for a consultation were included in this study (Fig. 1). Based on the year in which patients visited for a consultation (Fig. 1, upper section), the initial group of 4,899 patients with COVID-19 showed an increasing trend over the 3 years and a substantial increase in 2022. Over the study period, the rate of a “moderate or severe” diagnosis increased slightly from 34% in 2020 to 39% in 2021, but decreased extensively to 7% in 2022. Among the 1,353 patients included in the study (Fig. 1, lower section), the number of patients who visited our hospital for a consultation showed an upward trend from 24 to 1,219 over the 3 years, with a substantial increase to 1,219 in 2022; the rate of a diagnosis of “moderate or severe” increased from 24% in 2020 to 41% in 2021, but decreased substantially to 3% in 2022.

Table 1 shows the severity of COVID-19 infection by gender and age group. The proportion of “moderate or severe” COVID-19 diagnoses was 4.2% for women, 9.3% for men, and 6.8% for both genders. A significant relationship was observed between age and severity of infection: the higher the age, the higher the proportion of “moderate or severe” infections among men and total. A significant association was observed between gender and number of vaccinations (Table 2) for men, women, and both genders combined; the higher the number of vaccinations, the lower the rate of a diagnosis of “moderate or severe” infection.

**Table 1:** COVID-19 severity by gender and age over the entire period (2020–2022)

**gender**	**age**	**asymptomatic + ** **mild**	**moderate + ** **severe**	**total**
women	Teens + 20s + 30s	229	8	237
		96.6%	3.4%	100.0%

	40s	95	3	98
		96.9%	3.1%	100.0%

	50s	128	6	134
		95.5%	4.5%	100.0%

	60s	118	6	124
		95.2%	4.8%	100.0%

	70s	68	5	73
		93.2%	6.8%	100.0%

	80s + 90s	2	0	2
		100.0%	0.0%	100.0%

	total	640	28	668
		95.8%	4.2%	100.0%

men**	Teens + 20s + 30s	238	9	247
		96.4%	3.6%	100.0%

	40s	106	8	114
		93.0%	7.0%	100.0%

	50s	113	21	134
		84.3%	15.7%	100.0%

	60s	102	19	121
		84.3%	15.7%	100.0%

	70s	60	7	67
		89.6%	10.4%	100.0%

	80s + 90s	2	0	2
		100.0%	0.0%	100.0%

	total	621	64	685
		90.7%	9.3%	100.0%

total*	Teens + 20s + 30s	467	17	484
		96.5%	3.5%	100.0%

	40s	201	11	212
		94.8%	5.2%	100.0%

	50s	241	27	268
		89.9%	10.1%	100.0%

	60s	220	25	245
		89.8%	10.2%	100.0%

	70s	128	12	140
		91.4%	8.6%	100.0%

	80s + 90s	4	0	4
		100.0%	0.0%	100.0%

	total	1,261	92	1,353
		93.2%	6.8%	100.0%

*: P < 0.01 (chi-square test)

**: P < 0.001 (chi-square test)

**Table 2:** COVID-19 severity by gender and number of vaccinations over the entire period (2020–2022)

**gender**	**number of ** **vaccinations**	**asymptomatic + ** **mild**	**moderate + ** **severe**	**total**
women*	0 times	124	19	143
		86.7%	13.3%	100.0%

	once	5	0	5
		100.0%	0.0%	100.0%

	twice	305	7	312
		97.8%	2.2%	100.0%

	3 times or more	206	2	208
		99.0%	1.0%	100.0%

	total	640	28	668
		95.8%	4.2%	100.0%

men*	0 times	118	43	161
		73.3%	26.7%	100.0%

	once	2	0	2
		100.0%	0.0%	100.0%

	twice	309	14	323
		95.7%	4.3%	100.0%

	3 times or more	192	7	199
		96.5%	3.5%	100.0%

	total	621	64	685
		90.7%	9.3%	100.0%

total*	0 times	242	62	304
		79.6%	20.4%	100.0%

	once	7	0	7
		100.0%	0.0%	100.0%

	twice	614	21	635
		96.7%	3.3%	100.0%

	3 times or more	398	9	407
		97.8%	2.2%	100.0%

	total	1,261	92	1,353
		93.2%	6.8%	100.0%

*: P < 0.001 (chi-square test)

Table 3 shows the ORs for COVID-19 exacerbation according to the study factors after adjusting for confounding factors using backward stepwise regression. The explanatory variables extracted as factors that exacerbated COVID-19 severity were gender (OR [man vs. woman]: 2.533, 95% confidence interval [CI] 1.484–4.322); age (OR [50s vs. 10s, 20s, or 30s]: 4.858, 95% CI 2.319–10.177; OR [60s]: 9.738, 95% CI 4.355–21.777; OR [70s + 80s + 90s]: 8.327, 95% CI 3.224–21.507); and comorbid chronic lung disease (OR [‘yes’ vs. ‘no’]: 2.892, 95% CI 1.227–6.818). The explanatory variables extracted as factors that reduce the severity of COVID-19 were hospital consultation year (OR [2022, predominantly Omicron variant prevalent vs. 2020, predominantly Alpha variant prevalent]: 0.180, 95% CI 0.058–0.559); number of vaccinations (OR [2 doses vs. 0 or one doses]: 0.223, 95% CI 0.114–0.436; OR [≥3 doses vs. 0 or one doses]: 0.090, 95% CI 0.035–0.229); regular exercise (exercising ≥2 days/week ≥30 minutes each at an intensity that causes a slight sweat for ≥1 year) (OR [‘yes’ vs. ‘no’]: 0.458, 95% CI 0.242–0.866).

**Table 3:** Odds ratios of each factor for exacerbation$ of severity in patients with COVID-19#

				**95% confidence interval for ** odds ratio*				**95% confidence interval for ** odds ratio**		
**factor**	**Number of people**	**%**	odds ratio*	**lower limit**		**upper limit**	odds ratio**	**lower limit**		**upper limit**
gender										
woman	658	49.5%	1.000				1.000			
man	672	50.5%	2.547	1.401	-	4.629	2.533	1.484	-	4.322

age										
Teens + 20s + 30s	473	35.6%	1.000				1.000			
40s	207	15.6%	1.306	0.518	-	3.294	1.525	0.631	-	3.685
50s	266	20.0%	4.203	1.873	-	9.432	4.858	2.319	-	10.177
60s	241	18.1%	7.789	3.188	-	19.032	9.738	4.355	-	21.777
70s + 80s + 90s	143	10.8%	6.529	2.257	-	18.890	8.327	3.224	-	21.507

hospital consultation year										
2020	24	1.8%	1.000				1.000			
2021	109	8.2%	2.027	0.623	-	6.594	1.849	0.617	-	5.541
2022	1,197	90.0%	0.183	0.054	-	0.620	0.180	0.058	-	0.559

number of vaccinations										
0 times + once	305	22.9%	1.000				1.000			
twice	626	47.1%	0.190	0.093	-	0.390	0.223	0.114	-	0.436
3 times or more	399	30.0%	0.065	0.023	-	0.182	0.090	0.035	-	0.229

body mass index (BMI)										
BMI ≦ 18.5 (thin)	59	4.4%	0.429	0.068	-	2.693				
18.5 ≦ BMI < 25.0 (standard)	753	56.6%	1.000							
25.0 ≦ BMI < 30.0 (overweight)	347	26.1%	1.916	1.049	-	3.496				
30.0 ≦ BMI (obesity)	171	12.9%	1.921	0.888	-	4.155				

hypertension										
none	1,048	78.8%	1.000							
prevalent	282	21.2%	1.005	0.477	-	2.119				

diabetes										
none	1,241	93.3%	1.000							
prevalent	89	6.7%	1.956	0.721	-	5.305				

chronic lung disease										
none	1,228	92.3%	1.000				1.000			
prevalent	102	7.7%	2.716	1.106	-	6.665	2.892	1.227	-	6.818

chronic kidney disease										
none	1,327	99.8%	1.000							
prevalent	3	0.2%	0.000	0.000						

heart disease										
none	1,280	96.2%	1.000							
prevalent	50	3.8%	1.201	0.374	-	3.858				

malignant tumor										
none	1,316	98.9%	1.000							
prevalent	14	1.1%	8.582	1.542	-	47.767				

immunosuppressive state										
none	1,318	99.1%	1.000							
prevalent	12	0.9%	0.000	0.000						

dyslipidemia										
none	1,234	92.8%	1.000							
prevalent	96	7.2%	1.821	0.693	-	4.783				

having an evening meal 2 hours or less before bedtime 3 days or more per week										
no	888	66.8%	1.000							
yes	442	33.2%	0.871	0.484	-	1.567				

eating snacks and sweet beverages outside of breakfast, lunch, and dinner										
no	454	34.1%	1.000							
sometimes	635	47.7%	0.910	0.452	-	1.834				
every day	241	18.1%	0.702	0.268	-	1.842				

eating faster than others										
slow	115	8.6%	1.000							
usual	796	59.8%	0.760	0.295	-	1.957				
fast	419	31.5%	0.519	0.189	-	1.430				

skipping breakfast at least three times a week										
no	854	64.2%	1.000							
yes	476	35.8%	1.309	0.736	-	2.330				

walking for at least 1 hour per day or having equivalent physical activities										
no	817	61.4%	1.000							
yes	513	38.6%	1.413	0.814	-	2.451				

exercising at least 2 days per week for at least 30 minutes each session at an intensity that causes a slight sweat for at least 1 year										
no	949	71.4%	1.000				1.000			
yes	381	28.6%	0.454	0.233	-	0.886	0.458	0.242	-	0.866

frequency of drinking										
never	454	34.1%	1.000							
sometimes	635	47.7%	0.642	0.334	-	1.233				
every day	241	18.1%	0.717	0.322	-	1.594				

regular smoker										
no	1,031	77.5%	1.000							
yes	299	22.5%	0.903	0.465	-	1.754				

feeling refreshed after a night’s sleep										
no	410	30.8%	1.000							
yes	920	69.2%	1.033	0.595	-	1.794				

#: the number of subjects for analysis was 1,330 (23 were excluded due to partial missing values)

$: “moderate or severe” severity

odds ratio*: odds ratio by forced entry method for binomial logistic regression analysis

odds ratio**: odds ratio by backward stepwise method for binomial logistic regression analysis

## Discussion

To clarify the lifestyle factors that influence the exacerbation of COVID-19 severity, in this study we analyzed data for 1,353 patients with COVID-19 who visited our hospital and underwent a specific health checkup at our hospital before the date of their hospital consultation. We conducted a cross-sectional study with COVID-19 exacerbation as the objective variable and identified gender, age, and coexisting chronic lung disease as factors that exacerbate COVID-19 severity; year of the hospital consultation, number of vaccinations, and regular exercise were factors that reduced the severity. These results suggest important factors in establishing preventive measures against exacerbation of COVID-19 severity.

The factors gender, age, and coexisting chronic lung disease showed significant ORs (Table 3). Many studies have reported on the rate of severe disease by gender, and the rate of hospitalization, ICU cases, and deaths tend to be higher in men than in women [10, 11]. This has been attributed to differences in adaptive immune responses, effects of sex hormones on inflammatory processes, expression levels of angiotensin-converting enzymes, and lifestyle differences [12–14].

Several cohort studies of patients with COVID-19 have shown that older age is associated with greater disease severity [10, 11, 15]. Physiological changes associated with aging and comorbidities such as diabetes, hypertension, obesity, cardiovascular disease, and pulmonary disease—commonly seen in older adults—are thought to be the causes [16]. In addition, the increase in senescent immune cells observed with aging contributes not only to decreased host defenses, but also to an elevated inflammatory phenotype in immune dysfunction [17–19].

The main chronic lung diseases include chronic obstructive pulmonary disease, lung cancer, pneumonia, etc. The main cause and aggravating factor of these lung diseases is smoking [20]. Smoking habits did not show a significant OR in this study; however, an analysis of the amount of smoking conducted over a lifetime seems necessary. In any event, prevention of chronic lung disease appeared to reduce exacerbation of COVID-19 severity.

Factors that reduced the severity of COVID-19 showed significant ORs and included the year of the hospital consultation, number of vaccinations, and regular exercise (Table 3). The years 2020, 2021, and 2022 corresponded to the periods when mainly the Alpha, Delta, or Omicron variants, respectively, were prevalent. The OR for 2022 patients to 2020 patients showed a significant mitigation value, possibly due to the difference in the epidemic’s mutated strains found in each year. The Omicron mutant is significantly more infectious than the Alpha and Delta mutants are, but the rate of severe disease is low [21, 22]. The results of our study confirmed this point.

Compared to no vaccination, the ORs of two and three or more vaccinations showed significant reductions in incidence of moderate or severe COVID-19 infection. The preventive effect of vaccination against the Alpha, Delta, and Omicron variants, which were prevalent during the study period, has been clarified [23, 24]; however, the results of our study showed that vaccination was extremely effective in preventing exacerbation of severity. In studies conducted around the same time in Nara Prefecture, Japan [25, 26], more vaccinations were associated with a lower risk of COVID-19-related health outcomes, not only for Delta variants but also for Omicron variants. This was consistent with the results regarding the effectiveness of vaccination shown in this study.

Patients who exercised regularly showed significantly reduced severity of disease compared to patients who did not exercise regularly. Elevated hemoglobin A1c levels have been associated with a worse prognosis of COVID-19 and hyperglycemia is a factor in the severity of COVID-19 [27, 28]. These studies suggest a mechanism by which regular exercise may improve glucose metabolism and thus reduce the exacerbation of COVID-19 severity.

## Limitations

**Fig. 2:**
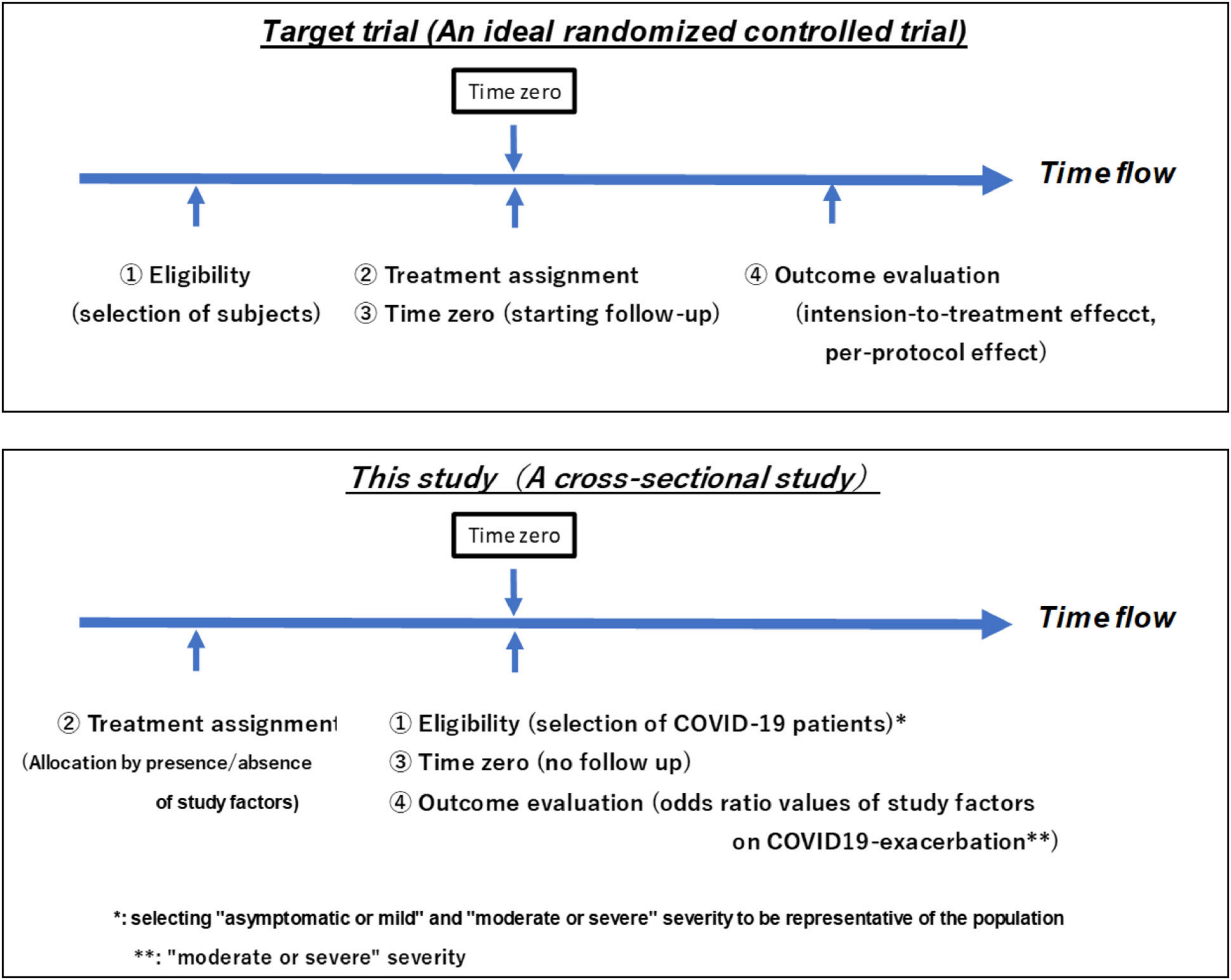
Time relationship among main items of target trial emulation framework in this study

The limitations of this research method were examined using a target trial emulation framework which examined four items: (1) eligibility, (2) treatment assignment, (3) time zero (timing to start follow-up), and (4) outcome evaluation (per-protocol effect, intention-to-treat effect) [29, 30]. In a target trial (an ideal randomized controlled trial), participants are first screened based on a series of eligibility criteria, assigned to an intervention group (treatment assignment), and then followed up (time zero). Finally, the outcomes are evaluated (Fig. 2). In this study, treatment assignment (allocation by presence/absence of the explanatory variables) was performed first, then eligibility (selection of patients with COVID-19), time zero (no follow up), and outcome evaluation (ORs of study factors on COVID-19 exacerbation calculated) were implemented simultaneously at time zero.

Eligibility for inclusion in this study may have introduced selection bias. We calculated the ORs of study factors on COVID-19 exacerbation as outcome evaluations; however, two conditions must be met in order for the OR to approximate the Relative Risk [31]. The first condition is that the frequency of occurrence of “moderate or severe” severity must be low, and the second condition is that the samples selected for the study must be representative of the “asymptomatic or mild” and “moderate or severe” populations. Regarding the first condition, the incidence of COVID-19 itself was low, and the proportion of “moderate or severe” severity was 6.8% of the total, which seemed to satisfy the first condition (Table 1). Regarding the second condition, whether the samples selected for the study were selected to represent the “asymptomatic or mild” and “moderate or severe” populations is unclear, and the possibility of selection bias cannot be denied. In the area in which the study was conducted—in 2022, comprising one city, one town, seven villages, and approximately 102,000 people—two hospitals, including ours, provided beds for patients with infectious diseases. These two hospitals account for the majority of COVID-19 cases in the region. The 1,353 study participants were extracted from 4,899 patients who visited our hospital. The distribution of severity among the 4,899 cases seems likely to be representative of that of the local population. As the severity distribution of the 4,899 patients who visited our hospital and the severity distribution of the extracted 1,353 patients was similar (Fig. 1), the extent of the selection bias seemed to be small.

Information on explanatory variables (treatment assignment) were obtained from the most recent health checkups before the hospital consultation for COVID-19. A major problem with cross-sectional studies is that whether the factors precede the outcome is unclear. Since the capture of factors in this study was obtained from health checkups performed prior to the incidence of COVID-19, the factors clearly preceded the outcome events. The duration for which the explanatory variables were maintained from the time of the health checkup to the evaluation of the COVID-19 exacerbation was unclear, making per-protocol analysis impossible. The explanatory variables were analyzed under the assumption that they were maintained throughout the observation period. The method of this study seems to correspond to the intention-to-treat analysis of a target trial. Since the validity of the intention-to-treat analysis in a target trial has been confirmed, the analysis in this study appears to be valid [32].

## Conclusion

We conducted a cross-sectional study of 1,353 patients with COVID-19 to clarify the lifestyle factors that influence exacerbation of COVID-19 severity. Exacerbating factors included gender, age, and chronic lung disease, while mitigating factors included which year patients visited our hospital for consultation, the number of vaccinations patients had been administered, and patient regular exercise. The results suggest that vaccination, regular exercise, and prevention of chronic lung disease are important factors for preventing exacerbation of COVID-19 severity.

## Abbreviations

CI: confidence interval
COVID-19: coronavirus disease 2019
COVID-19 exacerbation: severity classification “moderate or severe”
OR: odds ratio

## Declarations

### Availability of data and material

The data analyzed in this study are not publicly available.
